# Effect and safety of Neiguan acupoint injection with astragalus injection for chronic heart failure with qi-deficiency and blood-stasis syndrome: a randomized controlled trial

**DOI:** 10.3389/fmed.2026.1836620

**Published:** 2026-05-28

**Authors:** Dongmei Gou, Maomei He, Jiaquan Zhuo

**Affiliations:** Department of Cardiology, Traditional Chinese Medicine Hospital of Renshou County, Renshou, China

**Keywords:** astragalus injection, chronic heart failure, Neiguan acupoint, qi-deficiency and blood-stasis syndrome, renin-angiotensin-aldosterone system

## Abstract

**Objective:**

To investigate the effect and safety of astragalus injection at Neiguan (PC6) for chronic heart failure (CHF; HF) with qi-deficiency and blood-stasis syndrome, and its effect on the renin-angiotensin-aldosterone system (RAAS).

**Methods:**

A randomized, double-blind, placebo-controlled, parallel-group trial was conducted. Patients with CHF presenting with qi-deficiency and blood-stasis syndrome were randomly assigned in a 1:1 ratio to either the experimental group or control group using a random number table. The experimental group received conventional standard therapy plus astragalus injection at PC6, whereas the control group received conventional standard therapy plus placebo. Traditional Chinese Medicine (TCM) syndrome scores, B-type natriuretic peptide (BNP) levels, left ventricular ejection fraction (LVEF), Minnesota Living with Heart Failure Questionnaire (MLHFQ) scores, 6-min walk distance (6MWD), New York Heart Association (NYHA) functional class, plasma renin (PRA), angiotensin II (Ang II), aldosterone (ALD), were compared between groups at baseline and post-treatment. 3-month major adverse cardiac events (MACE), including all-cause mortality and HF-related readmission. Adverse events and safety indicators were also monitored.

**Results:**

A total of 200 patients were included in the final analysis, 100 in each group. Baseline characteristics were not significantly different between the two groups (*p* > 0.05), except for the grouped distribution of patients stratified by LVEF level. Compared with the control group after treatment, the experimental group exhibited significantly reductions in TCM syndrome scores, BNP, MLHFQ scores, NYHA functional class, PRA, Ang II and ALD (*p* < 0.05), as well as significantly greater increases in LVEF and 6MWD (*p* < 0.05). There was no significant difference in readmission between the two groups (*p* > 0.05). No adverse events were observed in either group.

**Conclusion:**

Acupoint injection at PC6 with astragalus injection combined with conventional standard therapy can effectively improve cardiac function, exercise tolerance and quality of life in patients with CHF with qi-deficiency and blood-stasis syndrome. It is a safe and effective adjuvant treatment for CHF.

**Clinical trial registration:**

Registration number: ITMCTR2026000404. https://itmctr.ccebtcm.org.cn/mgt/project/user/user-project-view/1be0197d-9db9-434d-a2d1-fa4e5bccbc44

## Graphical abstract

**Figure:**
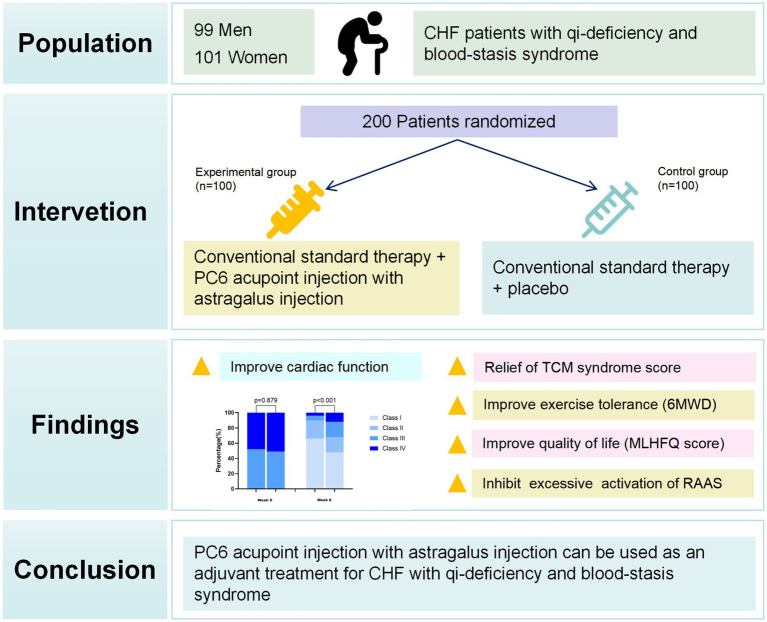
RCT: Neiguan (PC6) acupoint injection with astragalus injection for chronic heart failure with qi-deficiency and blood-stasis syndrome.

## 1 Introduction

Population aging in China has led to a steady increase in chronic cardiovascular diseases, including coronary heart disease, hypertension, and diabetes, consequently raising the prevalence of HF as the end-stage phenotype of cardiovascular disorders, which has resulted in a corresponding rise in the number of HF patients, making HF a major public health challenge in China (1). Epidemiological data show that the global prevalence of HF in adults ranges from 1 to 3%. A national survey in 2017 reported approximately 12.1 million HF patients in China, with a prevalence of 1.1% among individuals aged ≥25 years and approximately 2.97 million new cases annually. HF is associated with a 16.4% one-year readmission rate, 16.4% cardiovascular mortality, and 8.5% all-cause mortality (2). The large CHF population is associated with substantial economic pressure due to high medical costs. Globally, the annual expenditure for the diagnosis and treatment of HF reaches $65 billion (2). In China, the average annual number of hospitalizations per CHF patient is 3.3, with 59.8% of patients being hospitalized twice or more per year. The mean annual hospitalization cost per patient is ¥29,746, and the average annual outpatient cost is ¥6,023 (2). CHF is characterized by high incidence, high mortality, and high readmission rates (2). Thus, alleviating the socioeconomic burden of CHF has become an urgent public health priority.

The RAAS plays an essential role in the mechanism of CHF. Numerous studies have shown that the RAAS plays a cardinal role in the mechanism of HF (3, 4). Excessive RAAS activation promotes salt and water retention, peripheral vasoconstriction, and left ventricular hypertrophy, ultimately leading to cardiac and renal fibrosis. Ang II impairs sodium and water excretion, while aldosterone induces myocardial fibrosis, vascular damage, and baroreceptor dysfunction (5). Therefore, inhibiting abnormal RAAS activation represents a key therapeutic target in CHF.

Currently, the primary drugs used to inhibit the RAAS include Angiotensin-Converting Enzyme Inhibitors (ACEI), Angiotensin II Receptor Blockers (ARB), Mineralocorticoid Receptor Antagonist (MRA) and the Angiotensin Receptor Neprilysin Inhibitor (ARNI) (6). Drug treatments for HF aim to alleviate symptoms by targeting the neurohormonal system’s maladaptive responses and improving heart function (7). Key medications, such as ACEI and beta-adrenergic receptor blockers (*β*-blockers), are considered first-line therapy for HFrEF, working to reduce complications and enhance longevity (8). Additionally, ARNI combines an ARB with a neprilysin inhibitor to further manage the condition (9).

However, although these drugs have shown some efficacy in the stabilization of the symptomatology of HF, significant limitations and inadequacies remain (10). For instance, ACEI may cause hypotension, mild azotemia, nonproductive cough, angioedema and potassium retention (4). MRA may lead to hyperkalemia (11). Statistics reveal that despite continually optimised treatment protocols for HF, the incidence and mortality of CHF remain high (2).

Traditional Chinese medicine exhibits characteristics such as multi-targeted, multi-pathway mechanisms and high safety profiles in treating cardiovascular diseases, with significant clinical efficacy (12, 13). Research has revealed that TCM can regulate both mRNA and protein expression of factors such as Bax, cleaved caspase-3, cleaved caspase-9, Cyt C, TNF-*α*, IL-6, and Bcl-2 through classical pathways including the death receptor pathway, mitochondrial pathway, and endoplasmic reticulum pathway (14). Concurrently, it can inhibit apoptosis and inflammatory responses by modulating multiple signaling pathways including TGF-*β*1/Smad3, Nrf2/Keap1/HO-1, and PI3K/Akt, thereby protecting cardiomyocytes, improving cardiac function, and delaying the progression of CHF (15).

Qi-deficiency and blood-stasis syndrome is the primary pattern type in the clinical stage of CHF (16). The therapeutic principle centers on tonifying qi and invigorating blood circulation (17).

PC6, a key point of the Pericardium Meridian, regulates qi and blood flow, relieves chest distress, and promotes blood circulation (18). Astragalus injection exerts potent effects of tonifying qi, activating blood, and unblocking collaterals (19, 20). Astragalus can tonify qi, while stimulating the PC6 invigorates blood circulation. The combined use of both achieves the dual effect of tonifying qi and invigorating blood circulation. Based on this, we hypothesize that acupoint injection of astragalus injection at PC6 will yield significant therapeutic benefits for CHF with qi-deficiency and blood-stasis syndrome.

Meanwhile, research has demonstrated that astragalus can alleviate the excessive activation of the RAAS (21). Our preliminary animal studies demonstrated that acupuncture at PC6 reduces BNP levels in rabbits with CHF, improves cardiac function, suppresses activation of the RAAS and catecholamine systems, and exerts beneficial effects on myocardial remodeling processes (22). Additionally, findings from other animal studies also support our earlier research results. The study by Gao et al. (2021) demonstrated that electroacupuncture stimulation of PC6 significantly reduced serum and brain tissue levels of PRA, Ang II, and ALD in rats, effectively maintaining the stability of the renin-angiotensin system in tail-suspension-simulated weight-loss rats for 7 days (23). Tao et al. (2024) investigated the effects of acupuncture at PC6 on myocardial histopathology in hypertrophic mice (24). Results demonstrated that acupuncture at PC6 slowed heart rate, inhibited myocardial fibrosis, reduced inflammatory responses, improved myocardial hypertrophy, and delayed myocardial damage. Sun et al. (2021) found that electroacupuncture at PC6 improved cardiac function and reduced myocardial cell apoptosis in rats with acute myocardial ischemia, potentially through regulating inflammatory factors in PC6 region (25).

Thus, both PC6 stimulation and astragalus injection exert effects the RAAS. Therefore, we also hypothesize that the efficacy of astragalus injection administered at PC6 for treating CHF may be related to its suppression of excessive RAAS activation. However, the efficacy of astragalus injection administered via acupoint injection at PC6 for treating CHF with qi-deficiency and blood-stasis syndrome remains unclear. No systematic study has previously investigated the combined effects of PC6 stimulation and astragalus injection on CHF with qi-deficiency and blood-stasis syndrome. This randomized, double-blind, placebo-controlled trial investigates the clinical efficacy and underlying mechanisms of PC6 acupoint injection with astragalus injection in CHF patients with qi-deficiency and blood-stasis syndrome. This study aims to provide adjuvant therapeutic strategies for CHF and enhance the quality of life for affected patients.

## 2 Materials and methods

### 2.1 Sample collection

**Figure 1:**
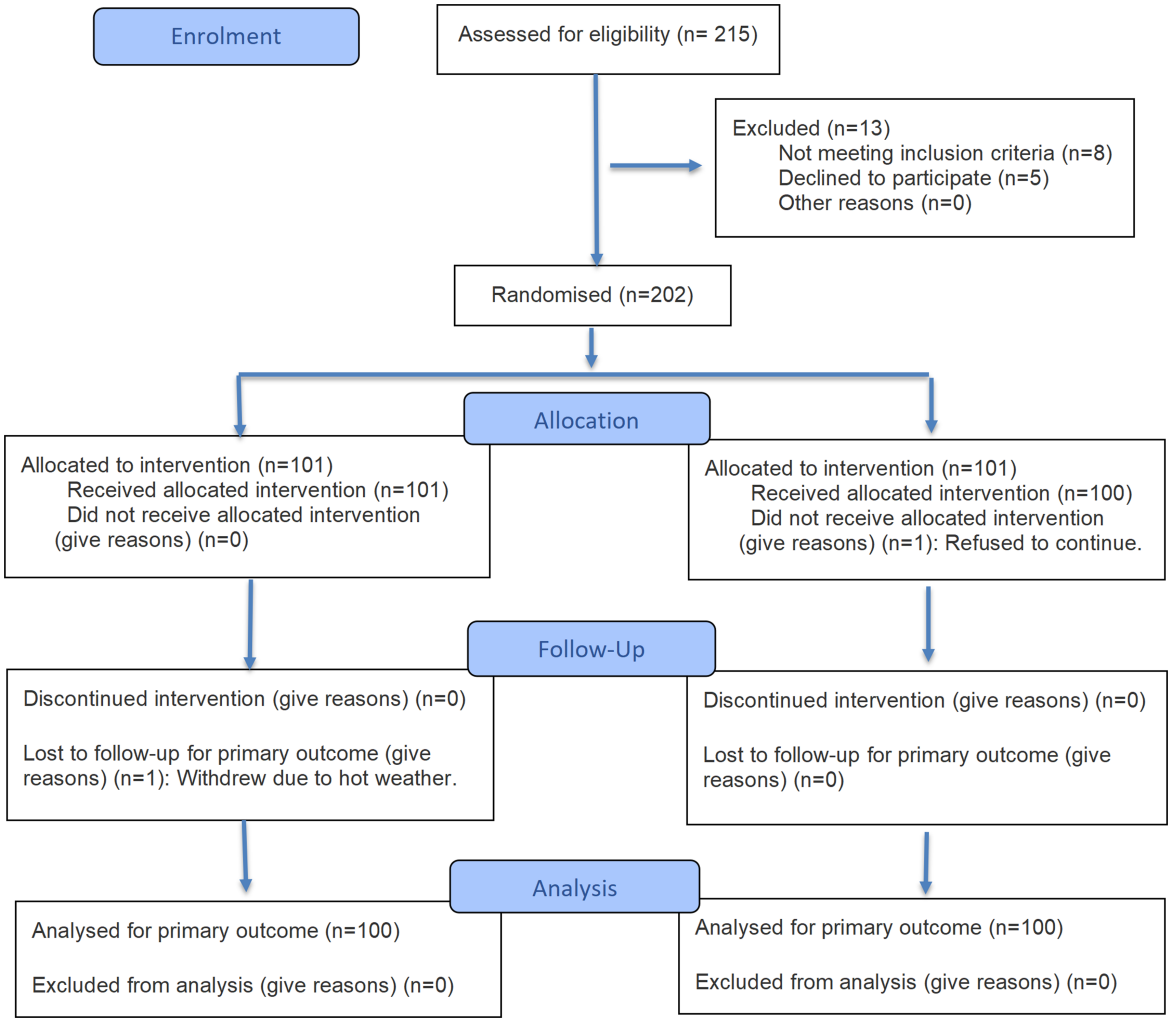
CONSORT 2025 flow diagram of participant progress through the trial.

All clinical samples were collected from the Traditional Chinese Medicine Hospital of Renshou County between May 2024 and August 2025. The flow of participants throughout the trial is presented in Figure 1 (26).

### 2.2 Study design

This study was designed as a randomized, double-blind, placebo-controlled, parallel-group trial.

Randomization: Participants were randomly assigned to two groups in a 1:1 ratio using a random number table. This study adopted the block randomization method for group allocation, with a block size of 4 (modify as needed). Age and severity of the disease were set as stratification factors (replace with actual stratification factors). The random grouping sequence was strictly generated by the random number table method, compiled and kept by an independent researcher. Participants of the RAAS substudy were consecutively enrolled in chronological order from the randomized cohort of the main trial, without additional independent randomization or stratification based on baseline RAAS inhibitor medication.

Allocation concealment: Allocation concealment was implemented using sealed opaque envelopes marked with unique serial numbers. The random sequence was generated and maintained by an independent researcher not involved in enrollment or treatment. The corresponding envelope was opened to confirm the group allocation only after participants completed the baseline assessment.

Double-blind: Both investigators (except for the acupoint injection administrators) and patients were blinded to group allocation. A blinding success assessment will be performed at the end of the trial. All participants and acupoint injection operators will be asked to guess the group allocation. This procedure is designed to verify the adequacy of the double-blind design.

Assessor blinding: Outcome assessors were unaware of group assignments, and all laboratory tests were performed by an independent third party.

Placebo: Sterile water for injection was used as placebo instead of astragalus injection. Patients were not informed of the appearance of the study medication before enrollment. No obvious difference in injection sensation or odor could be perceived between the two preparations. Participants were unfamiliar with the standard color characteristics of astragalus injection, and the medication preparation and injection procedures were performed out of the participant’s sight to avoid visual distinction. Participants were arranged with staggered visit time to minimize mutual contact. No guidance or discussion related to medication sensation and appearance was encouraged during the study visit.

Unblinding: Emergency unblinding was permitted only in the event of serious adverse events.

Acupoint injections were administered by independent investigators who did not participate in data collection or statistical analysis. The study manager did not directly participate in patient treatment or follow-up, but was responsible for data summary at the end of the study. The study manager had access to all patient data, while other researchers were not permitted to obtain information on individual treatment allocation.

This study was approved by the Ethics Committee of the Traditional Chinese Medicine Hospital of Renshou County (approval number: 2024042517) and registered in the International Traditional Medicine Clinical Trial Registry Platform (registration number: ITMCTR2026000404). Written informed consent was obtained from all participants prior to enrollment.

### 2.3 Sample size calculation

Sample size estimation formula for comparing two independent sample means: $n=\frac{2[{\left(z_{1-\alpha /2}+Z_{1-\beta }\right)}^{2}\times {\sigma_{\rho }}^{2}]}{\delta^{2}}$ . The sample size calculation was based on the primary outcomes of previous study. With a two-sided *α* = 0.05 and a power (1 − *β*) of 0.9, and assuming an equal sample size ratio of 1:1 between the experimental and control groups. Based on the data of BNP, 6MWD, PRA, Ang II, ALD, and LVEF from previous studies, the minimum required sample sizes for each group were calculated to be 58, 75, 11, 2, 18, and 76, respectively (22, 27, 28). To ensure adequate statistical power for all outcome measures, the largest calculated sample size was adopted as the final sample size. Considering a potential 20% dropout rate, at least 95 subjects were needed in the experimental group and 94 in the control group, totaling a minimum sample size of 190. Given the diversity of the intervention factors, to ensure patient compliance and data completeness, we planned to recruit 200 patients in total. For the RAAS substudy, after considering the dropout rate, at least 22 participants per group were required.

### 2.4 Diagnostic criteria

#### 2.4.1 Diagnostic criteria for HF

The diagnostic criteria were adopted from the Chinese Guidelines for the Diagnosis and Treatment of Heart Failure 2024 (29).

#### 2.4.2 Diagnostic criteria for qi-deficiency and blood-stasis syndrome in TCM

The diagnostic criteria for qi-deficiency and blood-stasis syndrome were referenced from the Guidelines for Chinese Medicine Diagnosis and Treatment of Chronic Heart Failure 2022 (17).

*Major symptoms*: shortness of breath/wheezing, fatigue, palpitations.

*Minor symptoms*: lassitude and reluctance to speak, easy fatigue with activity; Spontaneous sweating without obvious cause during the daytime, aggravated by activity; Low voice volume; Dark complexion or purplish lips.

*Tongue and pulse manifestations*: purplish-dark tongue (or with ecchymosis, petechiae, or tortuous and purple sublingual veins), normal tongue size, white tongue coating, deep, thready or weak pulse.

The syndrome is diagnosed when ≥2 major symptoms and ≥2 minor symptoms are present, together with the corresponding tongue and pulse manifestations.

### 2.5 Inclusion and exclusion criteria

#### 2.5.1 Inclusion criteria

Patients were eligible if they met all of the following criteria:

- Aged between 18 and 90 years, with no gender restrictions;
- Met the diagnostic criteria for CHF; (29).
- Met the diagnostic criteria for Qi-deficiency and blood-stasis syndrome in TCM; (17).
- Voluntarily signed the informed consent form.

#### 2.5.2 Exclusion criteria

Patients were excluded if they met any of the following conditions:

- Known allergy to astragalus injection;
- Patients with CHF who have undergone implantation of a cardiac resynchronization therapy device or implantation of a cardioverter-defibrillator within the preceding 12 weeks, or who are scheduled to undergo such device implantation within the next 12 weeks;
- Acute coronary syndrome within the past month, or cases complicated by cardiogenic shock, acute myocarditis, uncontrollable malignant arrhythmias, hypertrophic obstructive cardiomyopathy, constrictive pericarditis, cardiac tamponade, severe valvular disease requiring surgical intervention, or pulmonary embolism;
- Severe hepatic or renal insufficiency, anaemia, autoimmune disorders, or malignant tumours;
- Patients with concomitant cardiogenic shock or impaired consciousness;
- Women planning to conceive, pregnant women, and breastfeeding women;
- Participation in other studies within the past 2 months;
- Received any TCM treatment within the 2 weeks prior to enrolment.

### 2.6 Intervention

#### 2.6.1 Group treatment and medications

All patients received standard treatment in accordance with the 2023 ESC Guidelines for the Diagnosis and Treatment of Acute and Chronic Heart Failure (6). Dosage of all medications was adjusted according to each patient’s heart rate, blood pressure, fluid intake and output, and fluid retention status (30). On this basis, the experimental group was additionally administered astragalus injection at PC6, while the control group received sterile water for injection as placebo control. All patients received 8 weeks of intervention. Detailed information on the medications used in this study is presented in Table 1. All study medications were uniformly provided by the research team.

**Table 1:** Details of study medications.

Drug type	Drug name	Dosage and administration	Drug specification	Manufacturer	Approval number
ACEI/ARB/ARNI	Amlodipine Besylate and Benazepril Hydrochloride Tablets (II)	5 mg; 10 mg orally once daily	5 mg; 10 mg	Chengdu Pharmaceutical Stock Co., Ltd.	H20090309
	Valsartan Tablets	40 mg orally once daily	40 mg	Changzhou Siyao Pharmaceuticals Co., Ltd.	H20010823
	Sacubitril Valsartan Sodium Tablets	100 mg orally twice daily	100 mg	Novartis Pharma Schweiz	HJ20170363
*β*-blockers	Metoprolol Tartrate Tablets	12.5 mg orally twice daily	25 mg,	Changzhou Siyao Pharmaceuticals Co., Ltd.	H32025169
MRA	Spironolactone Tablets	20 mg orally twice daily	20 mg	Hangzhou Minsheng Pharmaceutical Co., Ltd.	H33020070
SGLT2i	Dapagliflozin Tablets	10 mg orally once daily	10 mg	Hunan Jiudian Pharmaceutical Co., Ltd.	H20249826
Diuretic	Furosemide Tablets	20 mg, orally twice daily	20 mg	Tianjin Lisheng Pharmaceutical Co., Ltd.	H12020163
Chinese herbal injection	Astragalus Injection	1 mL, injected at PC6	10 mL	Heilongjiang ZBD Pharmaceutical Co., Ltd.	Z23020782
Placebo control	Sterile Water for Injection	1 mL, injected at PC6	2 mL	Sinopharm Group Rongsheng Pharmaceutical Co., Ltd.	H41024923

SGLT2i, Sodium-glucose cotransporter 2 inhibitor.

#### 2.6.2 Acupoint injection procedure

**Figure 2:**
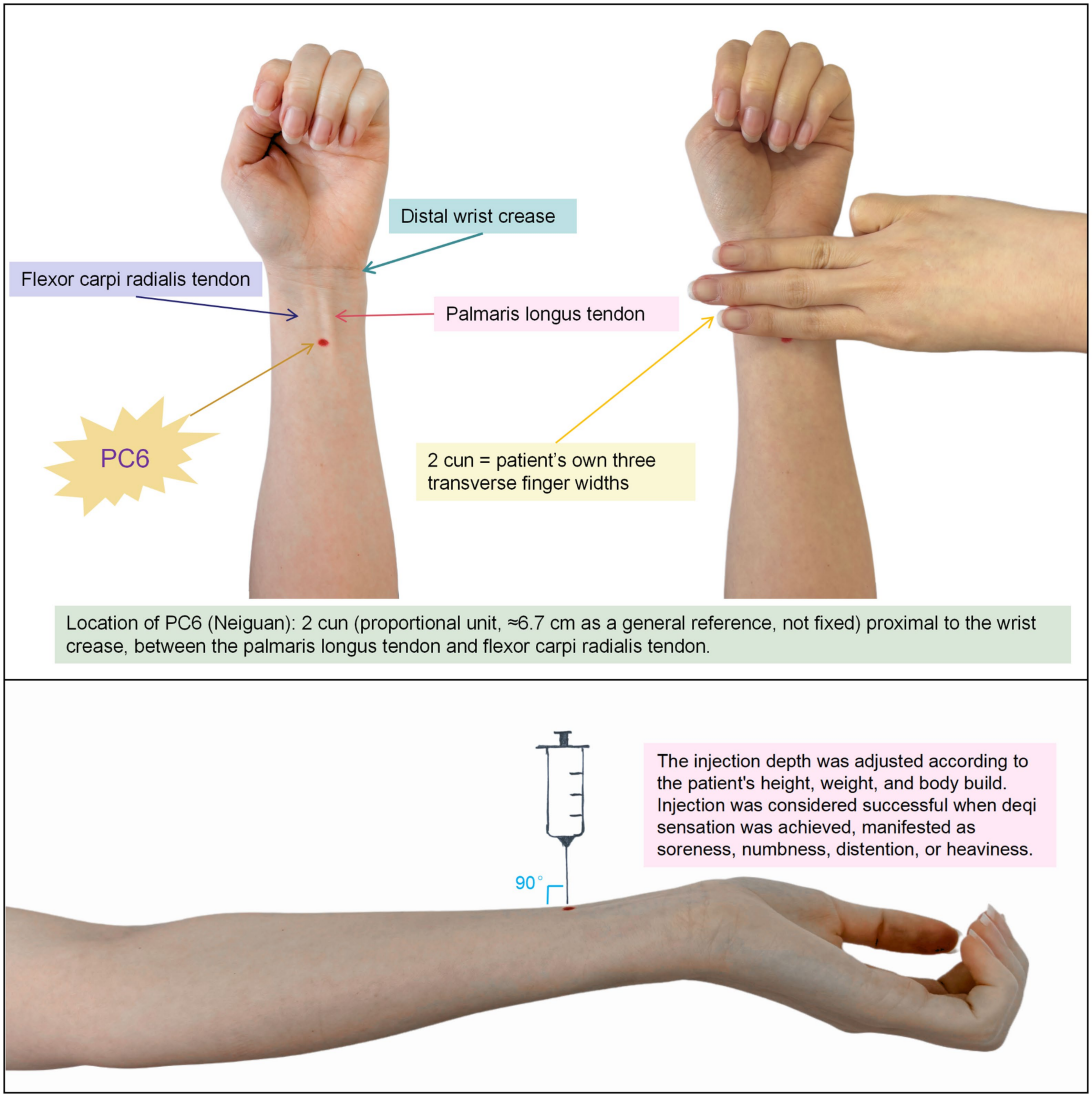
Schematic diagram of the location and acupuncture protocol at PC6 acupoint. The photograph shows the anatomical location of PC6 on the author’s own forearm, marked with a red dot. Considering individual differences, we use the patient’s own three transverse finger widths for acupoint location in clinical practice, which corresponds to the conventional Chinese proportional measurement of 2 cun. The schematic injection path and parameters are illustrated; all figures are original and copyright-free. No patients or identifiable individuals are involved. Since the image uses the author’s own body, ethical approval is not required.

1 mL of undiluted astragalus injection was administered bilaterally at PC6 acupoints. No further dilution or preparation was required prior to administration. The needle was inserted perpendicularly to the skin surface. The injection depth was adjusted according to the patient’s height, weight, and body build. Injection was considered successful when deqi sensation was achieved, manifested as soreness, numbness, distention, or heaviness. The dosage, administration frequency, and treatment duration for acupoint injection were optimized based on published clinical trials and routine clinical practice (31, 32). The procedure is illustrated schematically in Figure 2.

The overall intervention period was set as 8 weeks, consisting of three treatment courses. Each course included 5 acupoint injection sessions administered once every other day, with a 10-day interval between adjacent courses. To accommodate participants’ practical follow-up arrangement, a flexible time window of 1–2 days was allowed for each course schedule. All planned interventions were required to be completed within the 8-week study period. Each participant was scheduled to receive a total of 15 acupoint injection sessions. All treatment procedures were performed by qualified TCM physicians, and all session arrangements, time adjustments and protocol deviations were strictly documented throughout the trial.

#### 2.6.3 Placebo control

Sterile water for injection was used as placebo, with the same dosage, administration route, frequency, and total treatment duration as those in the experimental group. The injection site, needling depth, manipulation procedure, and operating duration were strictly consistent with those of the experimental group. No intentional induction of deqi sensation was performed in the placebo group.

#### 2.6.4 Monitoring of treatment adherence

A rigorous adherence monitoring system was implemented throughout the trial. All acupoint injections were performed by designated qualified TCM physicians. The implementation date, treatment course node, acupoint prescription and operator information were recorded in detail after each session, with dual signatures of both the participant and the operator for confirmation. Each participant was provided with an individualized treatment schedule at enrollment, clarifying the time arrangement of each course, interval period and the deadline for completing all interventions.

Participants who failed to attend the appointed visit were actively followed up by telephone within 48 h to confirm the reasons and reschedule the treatment within the allowable time window. Cases that failed to complete the intervention beyond the flexible time window were recorded as missed sessions and protocol deviations. All missed injection cases, delayed visits, time window deviations and protocol violation events were fully documented and summarized.

### 2.7 Measurement indexes

Primary endpoints were defined as TCM syndrome score and BNP. Secondary endpoints included LVEF, MHLQ, 6MWD, NYHA functional class and MACE. Aspartate aminotransferase (AST) and creatinine (CRE) were defined as safety endpoints. RAAS-related analyses were conducted as an exploratory subgroup in a subset of participants only. Given that only two primary endpoints were included in this study with a limited number of endpoints, and the present research was designed as an exploratory clinical analysis, formal multiplicity adjustment was not performed.

#### 2.7.1 TCM syndrome scores

The scoring criteria for TCM syndromes were formulated with reference to the Guiding Principles for Clinical Research of New Chinese Medicines (Trial Implementation; Table 2) (33).

**Table 2:** Scoring criteria for TCM syndromes in patients with CHF.

Symptom	0 points	2 points	4 points	6 points
Wheezing	None	Occasional dyspnea/wheezing, mild, without affecting rest, activity, or sleep	Dyspnea/wheezing induced by mild exertion, without affecting sleep	Dyspnea/wheezing at rest, inability to lie flat, and severe sleep disturbance
Fatigue	None	Lassitude and poor vitality, capable of daily work and activities	Marked fatigue and systemic weakness, barely capable of daily activities	Severe exhaustion, unable to perform daily activities
Palpitations	None	Mild palpitations on exertion, no impact on daily work	Obvious palpitations on exertion, relieved by rest, barely functional	Palpitations at rest, unable to perform daily activities
Lassitude and reluctance to speak	None	Mild	Moderate	Severe
Spontaneous sweating	None	Occasional sweating with minimal volume	Frequent sweating with moderate volume	Profuse sweating throughout the day and night
Low voice	None	Slightly decreased volume	Moderately decreased volume	Severely decreased volume
Purplish lips	None	Mild	Moderate	Severe

#### 2.7.2 Laboratory assessments

Peripheral venous blood samples were collected from all participants. For RAAS analysis, blood samples were drawn from patients in a seated position, between 8:00 and 9:00 in the morning. Due to practical constraints and patient availability, plasma levels of PRA, Ang II, and ALD were measured in a random subgroup of 28 patients in each group. In contrast, serum levels of BNP, as well as AST and CRE, were determined in all patients. All blood samples were immediately transported to the clinical laboratory of our hospital for routine analysis. All laboratory measurements were performed by an independent third-party clinical laboratory, which remained blinded to the group allocation of this study. Detailed assay procedures were conducted strictly in accordance with the laboratory’s standard routine protocols. Blood specimens were analyzed individually rather than batch-processed. All pre-analytical handling procedures were strictly standardized and consistent between the two groups throughout the study.

#### 2.7.3 LVEF

Conventional cardiac color Doppler echocardiography was performed to measure LVEF in all patients before and after treatment. Echocardiography was performed using a Toshiba Aplio 500 ultrasound system equipped with a PST-30BT probe (3.0 MHz). All cardiac structural and functional parameters were measured strictly in accordance with the standardized recommendations of the American Society of Echocardiography (34). All examinations were completed by a single independent sonographer who was blinded to both study group allocation and follow-up timepoints. Given that all measurements were interpreted by the same sonographer, inter-reader variability was not applicable, and no repeated offline reading was performed to assess intra-reader variability.

#### 2.7.4 MLHFQ

A Chinese translation of the MLHFQ was adopted. Patients scored themselves before and after 8 weeks of treatment, and the scores were calculated based on total score.

#### 2.7.5 6MWD

All patients underwent measurement of the 6MWD both prior to and following treatment. The 6-min walking test in this study was performed strictly in accordance with clinical standardized specifications. The specific testing protocol and quality control measures were described as follows.

##### 2.7.5.1 Test venue setting

The 6-min walking test was conducted in the routine public corridor of the ward, adopting the standard clinical shuttle walking mode with fixed markers in the corridor as turn-back points. All tests were arranged to avoid the peak flow of people in the ward, without deliberate closed clearance, and carried out based on the daily existing ward environment to minimize interference from irrelevant personnel and ensure the walking safety of subjects.

##### 2.7.5.2 Standardized training for assessors

All assessments of the 6-min walking test in this study were jointly completed by four clinicians after unified standardized systematic training. The training covered test operation process, shuttle timing rules, standardized instructions, test termination indicators, safety emergency plans and data recording standards. All assessors could participate in formal evaluation only after passing the unified theoretical and operational assessment.

##### 2.7.5.3 Unified instructions and practice walk requirements

Before the test, fixed standardized instructions were read to the subjects, informing them to walk back and forth at their maximum tolerable speed to cover the longest possible walking distance within 6 min. They could slow down or take a short standing rest during the test without forcing themselves. No special practice walk was arranged before the test; subjects were only asked to adapt briefly on site before the formal test.

##### 2.7.5.4 Repeated test rules

Both baseline and post-treatment 6-min walking tests for all subjects were arranged at the same fixed time every morning, with completely consistent walking routes and operation procedures. Each subject only underwent one formal test without repeated measurement; the final walking distance was uniformly calculated according to the number of shuttle laps and the length of conventional corridor sections in the ward.

##### 2.7.5.5 Timing relative to diuresis

Enrolled patients maintained their original routine oral diuretic treatment regimen throughout the study. All 6-min walking tests were uniformly conducted 4–6 h after routine diuretic administration. Both baseline and post-treatment follow-up tests strictly followed the same time interval standard to avoid fluctuations in body fluid volume status affecting test results and ensure the comparability of before-and-after data.

##### 2.7.5.6 Inter-rater reliability test

Before the formal test, a small sample of subjects was selected for the preliminary test. Four assessors independently completed the 6-min walking distance evaluation simultaneously in a double-blind manner. The intraclass correlation coefficient (ICC) was calculated to evaluate inter-rater reliability, and the result showed that ICC = 0.82, indicating excellent measurement consistency among the four assessors. The formal study strictly followed unified operational standards throughout to ensure stable and reliable evaluation results.

#### 2.7.6 NYHA functional class

Refer to the clinical implications of the New York heart association classification (35).

#### 2.7.7 MACE

3-month major adverse cardiac events (MACE), including all-cause mortality and HF-related readmission. Adverse events and safety indicators were also monitored. MACE were assessed during the 3-month follow-up period, defined as a composite endpoint, including all-cause mortality and HF-related readmission.

#### 2.7.8 Safety

Safety evaluation was performed throughout the entire study period, with standardized adverse event (AE) monitoring and definition protocols established in advance. Targeted solicited adverse events mainly included local injection-site reactions and systemic clinical symptoms. Local injection-site adverse events were defined as persistent pain, tenderness, bruising, swelling, redness, and infection at the injection site after completion of injection; transient physiological prickling pain during the injection procedure was regarded as a normal procedural response and not classified as a study-related adverse event. Systemic adverse events included dizziness, general fatigue, palpitations, nausea, and other unexplained systemic discomfort.

Adverse events were captured using a combination of active solicitation and passive spontaneous reporting. During each follow-up visit, investigators actively inquired about the above predefined adverse events through standardized verbal questioning, and participants were also instructed to spontaneously report any unexpected physical discomfort during the intervention period. The denominator for safety statistical analysis was defined as the total number of administered injections in this study. All adverse events were recorded in detail throughout the study, and any participant discontinuation due to intervention-related safety issues was documented comprehensively.

### 2.8 Statistical analysis

SPSS 29.0 (IBM Corp., Armonk, NY, USA) was used for statistical analysis (36). Normality tests were performed for all quantitative data. Normally distributed data were presented as mean ± standard deviation (SD). The paired *t*-test was applied for intra-group comparisons before and after treatment, and the independent two-sample *t*-test was used for inter-group comparisons between the experimental and control groups. Non-normally distributed data were described as median and interquartile range (IQR), with the Wilcoxon rank-sum test used for comparisons between two independent groups, and the Wilcoxon signed-rank test applied for paired intra-group comparisons. Categorical data were reported as frequencies or percentages (%) and compared using the chi-square (*χ*^2^) test. Ordinal ranked data at baseline and post-treatment were compared between the two independent groups using the Mann–Whitney *U* test. No intra-group paired intra-group comparisons were performed for ordinal data.

For the total TCM syndrome score, MLHFQ and 6MWD, multivariate linear regression was performed to assess the independent effect of intervention, with adjustment for age, gender, NYHA functional class and LVEF subgroup distribution. For BNP and LVEF, rank transformation was first applied to achieve approximate normality, followed by the same multivariate linear regression model adjusted for age, gender, NYHA functional class and LVEF subgroup distribution to control for potential confounding.

For normally distributed continuous data, subgroup comparisons were conducted using hierarchical linear regression, and interaction tests were performed using multiple linear regression model. For non-normally distributed continuous data, subgroup comparisons were performed using Mann–Whitney *U* test, and interaction tests were conducted using generalized linear model with Gamma distribution and log link function. When parameter estimation abnormality or model non-convergence occurred in the generalized linear model, rank-transformed two-way analysis of variance was used as an alternative method for interaction analysis.

All results are expressed as regression coefficients (B), 95% confidence intervals (95% CI), and *p*-values. A two-sided *p* < 0.05 was considered statistically significant.

## 3 Results

### 3.1 Baseline

A total of 202 patients with CHF presenting with qi-deficiency and blood-stasis syndrome were recruited. However, one patient in the experimental group was lost to follow-up due to the hot weather. In the control group, one patient voluntarily requested to withdraw without providing a reason, a total of 100 patients in the experimental group and 100 patients in the control group were included in the statistical analysis. The baseline characteristics of all patients are summarized in Table 3.

**Table 3:** Characteristics of the patients at baseline.

Characteristics	Experimental group (*n* = 100)	Control group (*n* = 100)	*t*/*χ*^2^/*z*	*p*
Age (years)	73.03 ± 11.21	75.14 ± 10.33	−1.384	0.168
Male	51	50	0.020	0.888
BMI (kg/m^2^)	22.88 ± 2.35	23.24 ± 3.04	−0.948	0.344
Smoking	14	15	0.040	0.841
LVEF (%)	63 (54–67)	63.5 (57.25–68)	−1.279	0.201
HFrEF	4	3	−0.153	0.041
HFmrEF	14	5		
HFpEF	82	92		
BNP (pg/mL)	325.26 (113.29–525.57)	266.86 (124.61–401.63)	−0.677	0.499
NYHA functional class				
Class I	0	0	−0.153	0.879
Class II	0	0		
Class III	52	49		
Class IV	48	51		
PLT, (×10^9^/L)	167.96 ± 62.51	183.59 ± 85.92	−1.471	0.143
Hypertension	47	56	1.621	0.203
Diabetes	24	17	1.503	0.220
Medications				
*β*-blockers	29	21	1.707	0.191
ACEI/ARB	13	23	3.388	0.066
ARNI	12	10	0.204	0.651
Diuretic	57	52	0.504	0.478
MRA	44	38	0.744	0.388
SGLT2	8	14	1.839	0.175

Data are presented as mean ± SD, *n*, or median (IQR). *T*-test, *χ*^2^ test, or Wilcoxon rank-sum test was used accordingly. Abbreviations: HFrEF, HF with reduced ejection fraction (LVEF ≤ 40%); HFmrEF, HF with mildly reduced ejection fraction (LVEF 41–49%); HFpEF, HF with preserved ejection fraction (LVEF ≥ 50%) (29).

All the remaining participants who completed the study finished all 15 scheduled acupoint injection sessions within the 8-week study period and the allowable flexible time window, with no missed injections, and the treatment adherence rate was 100%. No major protocol violations occurred throughout the trial. All participants who completed the trial strictly followed the research protocol, without unauthorized adjustment of intervention measures, over-time window visits, or prohibited combined medication. The median age was 74.1 years, and 50.5% were male. Patients had a high burden of comorbidities, including 103 patients (51.5%) with hypertension and 41 (20.5%) with diabetes. The median Body Mass Index (BMI) was 23.06. 29 (14.5%) patients had a history of smoking. Most patients were NYHA class III (101, 50.5%) and class IV (99, 49.5%). The median LVEF was 60.6%, including 7 patients with HFrEF (3.5%), 19 patients with HFmrEF (9.5%), 174 patients with HFpEF (87%). BNP was 325.26 (113.29–525.57) pg/mL in the experimental group and 266.86 (124.61–401.63) pg/mL in the control group at randomization. Besides, platelets (PLT) were 167.96 ± 62.51 × 10^9^/L in the experimental group and 183.59 ± 85.92 × 10^9^/L in the control group at randomization. The patients were well treated with guideline-recommended evidence-based HF therapies at baseline. Of those patients, 50 received beta blockers (25%), 36 received ACEI/ARB (18%), 44 received ARNI (22%), 109 received diuretics (54.5%), 82 received MRA (41%), and 22 received SGLT2 (11%). There were no significant differences in any of the baseline characteristics between the two groups (*p* > 0.05), except for the grouped distribution of patients stratified by LVEF level.

### 3.2 Effects on TCM syndrome scores

All of the TCM syndrome scores including wheezing, fatigue, palpitations, lassitude and reluctance to speak, spontaneous sweating, low voice and purplish lips were assessed before and after treatment. Before treatment, there was no statistically significant difference in every score between the experimental and control groups (*p* > 0.05). After treatment, the TCM syndrome scores in the experimental group were significantly lower than those in the control group (*p* < 0.05). The total points in the experimental group decreased from 23.42 ± 3.50 points to 11.72 ± 3.09 points, which was a statistically significant decrease (*p* < 0.05), while in the control group it decreased from 23.38 ± 3.43 points to 22.90 ± 2.75 points, which was not statistically significant (*p* > 0.05; Table 4). After adjusting for age, gender, NYHA functional class and LVEF subgroup distribution, the intervention remained independently associated with a significant improvement in the total TCM syndrome score (*B* = 11.210, 95%CI: 10.383 to 12.036, *p* < 0.001). These results indicate that the experimental treatment significantly improves the overall TCM syndromes in patients with CHF, whereas the control treatment shows no obvious effect on the TCM syndrome scores.

**Table 4:** Changes in TCM syndrome scores before and after treatment in two groups.

Subject	Time point	Experimental group	Control group	*t*	*p*
Wheezing (points)	Week 0	4.84 ± 1.11	4.80 ± 1.17	0.248	0.804
	Week 8	2.04 ± 1.02^*^	4.62 ± 1.01^**^	−17.912	<0.001^#^
Fatigue (points)	Week 0	3.00 ± 1.26	2.98 ± 1.26	0.113	0.910
	Week 8	1.50 ± 1.22^*^	2.96 ± 1.15^**^	−8.699	<0.001^#^
Palpitations (points)	Week 0	2.18 ± 1.18	2.14 ± 1.28	0.230	0.818
	Week 8	0.80 ± 1.06^*^	2.00 ± 1.30^**^	−7.135	<0.001^#^
Lassitude and reluctance to speak (points)	Week 0	4.22 ± 1.45	4.34 ± 1.24	−0.630	0.530
	Week 8	1.84 ± 1.20^*^	4.22 ± 0.89^**^	−15.944	<0.001^#^
Spontaneous sweating (points)	Week 0	2.22 ± 1.10	2.30 ± 1.83	−0.375	0.708
	Week 8	0.94 ± 1.04^*^	2.46 ± 1.10^**^	−10.050	<0.001^#^
Low voice (points)	Week 0	2.16 ± 1.01	2.10 ± 0.87	0.449	0.654
	Week 8	1.80 ± 0.60^*^	2.00 ± 0.64^**^	−2.283	0.024^#^
Purplish lips (points)	Week 0	4.80 ± 1.14	4.72 ± 1.08	0.509	0.611
	Week 8	2.80 ± 1.17^*^	4.64 ± 1.10^**^	−11.464	<0.001^#^
Total points (points)	Week 0	23.42 ± 3.50	23.38 ± 3.43	0.082	0.935
	Week 8	11.72 ± 3.09^*^	22.90 ± 2.75^**^	−27.050	<0.001^#^

*Comparison between Week 8 and Week 0 within the same group, *p* < 0.05; **Comparison between Week 8 and Week 0 within the same group, *p* > 0.05; #*p* < 0.05.

**Figure 3:**
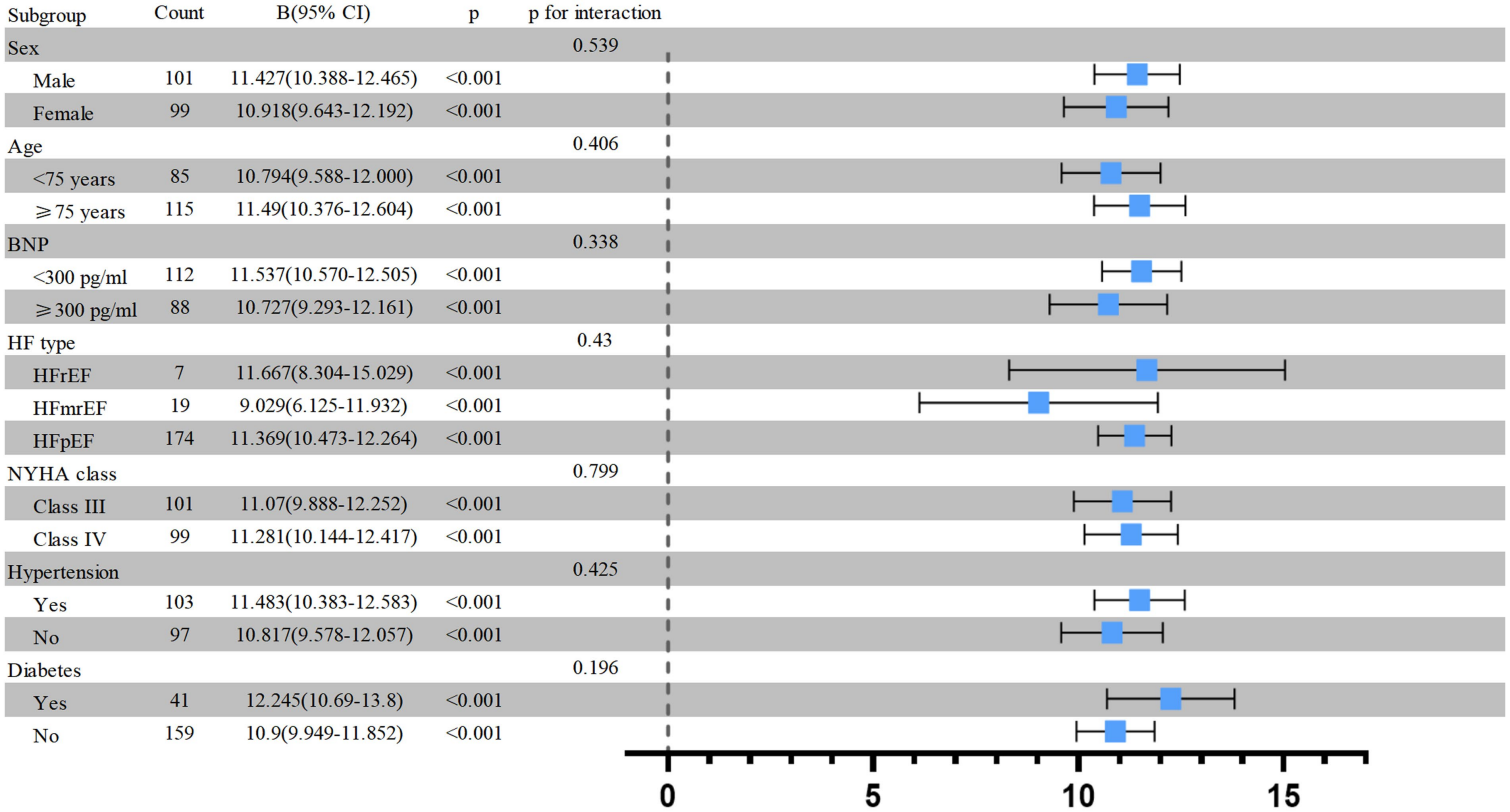
Forest plot of subgroup analysis for TCM syndrome score.

The results of subgroup analysis are presented in the forest plot of Figure 3. Subgroup analyses were performed stratified by gender, age, BNP, HF type, NYHA functional classification, hypertension and diabetes. In the subgroup analysis, all *p* values were <0.001, and all interaction *p* values were >0.05.

### 3.3 Effects on BNP

There was no significant difference in BNP between the two groups before treatment (*p* = 0.499). After treatment, a statistically significant inter-group difference was observed (*p* < 0.001). Intra-group comparisons showed that BNP was significantly decreased from 325.26 (113.29–525.57) pg/mL to 74.57 (34.28–166.73) pg/mL in the experimental group (*p* < 0.05). Similarly, BNP was also significantly reduced from 266.86 (124.61–401.63) pg/mL to 160.09 (87.57–295.54) pg/mL in the control group (*p* < 0.05; Table 5). After adjustment for age, gender, NYHA functional class and LVEF subgroup distribution, the intervention remained associated with significant improvements in rank-transformed BNP (B = 38.525, 95%CI: 23.871 to 53.178, *p* < 0.001). These findings indicate that both interventions effectively lower BNP levels in patients with CHF, with the experimental treatment yielding a significantly greater reduction.

**Table 5:** Changes in BNP, LVEF, MLHFQ, and 6MWD before and after treatment in two groups.

Subject	Time point	Experimental group	Control group	*t*/*z*	*p*
BNP (pg/mL)	Week 0	325.26 (113.29–525.57)	266.86 (124.61–401.63)	−0.677	0.499
	Week 8	74.57 (34.28–166.73)^*^	160.09 (87.57-295.43)^*^	−4.292	<0.001^#^
LVEF (%)	Week 0	63 (54–67)	63.5 (57.25–68)	−1.279	0.201
	Week 8	63 (57–67)^*^	58 (55-65)^*^	−2.859	0.004^#^
MLHFQ (points)	Week 0	64.55 ± 14.45	60.86 ± 17.97	1.601	0.111
	Week 8	49.72 ± 13.44^*^	58.24 ± 19.26^*^	−3.628	<0.001^#^
6MWD (meters)	Week 0	277.19 ± 74.29	265.73 ± 58.92	1.209	0.228
	Week 8	457.38 ± 99.66^*^	383.66 ± 120.91^*^	4.705	<0.001^#^

^*^Comparison between Week 8 and Week 0 within the same group, *p* < 0.05; #*p* < 0.05.

The results of subgroup analysis are presented in Table 6. Subgroup analyses were performed stratified by gender, age, BNP, HF type, NYHA functional classification, hypertension and diabetes. Notably, subgroup analyses stratified by LVEF revealed distinct statistical outcomes across subgroups, which were highly correlated with the imbalanced sample size distribution. Specifically, the number of enrolled patients was merely 7 in the HFrEF subgroup and 19 in the HFmrEF subgroup, in sharp contrast to 174 patients in the HFpEF subgroup. Such extreme sample size heterogeneity and high data dispersion directly led to inconsistent subgroup results: no statistically significant differences were observed in either the HFrEF or HFmrEF subgroups (all *p* > 0.05), whereas a highly significant difference was detected in the HFpEF subgroup (*p* < 0.001).

**Table 6:** Subgroup analysis for BNP.

Subgroup	Count	Median (IQR)	*p*	*p* for interaction
Sex				
Male	101	102.4300 (42.9550, 241.5450)	0.040	<0.001
Female	99	126.8200 (55.4900, 248.4500)	<0.001	
Age				
<75 years	85	112.0700 (47.8550, 182.0350)	<0.001	0.009
≥75 years	115	118.4000 (54.4900, 270.6900)	0.008	
BNP				
<300 pg/mL	112	81.725 (38.2625, 165.3775)	<0.001	<0.001
≥300 pg/ml	88	158.145 (89.895, 463.1275)	0.002	
HF type				
HFrEF	7	1,152.55 (142.9100, 5,000.00)	0.629	<0.001*
HFmrEF	19	184.92 (90.99, 429.45)	0.823	
HFpEF	174	97.52 (46.6875, 200.635)	<0.001	
NYHA class				
Class III	101	114.48 (57.88, 315.915)	0.002	<0.001
Class IV	99	114.68 (44.76, 226.86)	0.002	
Hypertension				
Yes	103	119.17 (48.05, 270.69)	0.037	0.089
No	97	111.85 (50.145, 198.065)	<0.001	
Diabetes				
Yes	41	91.55 (29.91, 171.96)	0.085	<0.001
No	159	119.17 (54.49, 284.34)	<0.001	

^*^Rank-transformed two-way analysis of variancexingbie.

There was no significant interaction between group and hypertension subgroup for BNP (*p* > 0.089), indicating consistent intervention effects across hypertension strata. Whereas a significant interaction was detected in the sex, age, BNP, HF type, NYHA class and diabetes subgroup (*p* < 0.05), suggesting that age, BNP, HF type, NYHA class, and diabetes stratification modified the intervention effect on BNP levels.

### 3.4 Effects on LVEF

No significant between-group difference in LVEF was observed at baseline (*p* = 0.201). After treatment, LVEF was significantly higher in the experimental group than in the control group (*p* = 0.004). Intra-group comparisons revealed that LVEF was significantly increased from baseline to week 8 in both the experimental and control groups (*p* < 0.05; Table 5). After adjustment for age, gender, NYHA functional class and LVEF subgroup distribution, the intervention remained associated with significant improvements in rank-transformed LVEF (*B* = −30.548, 95%CI: −44.286 to −16.810, *p* < 0.001).

**Figure 4:**
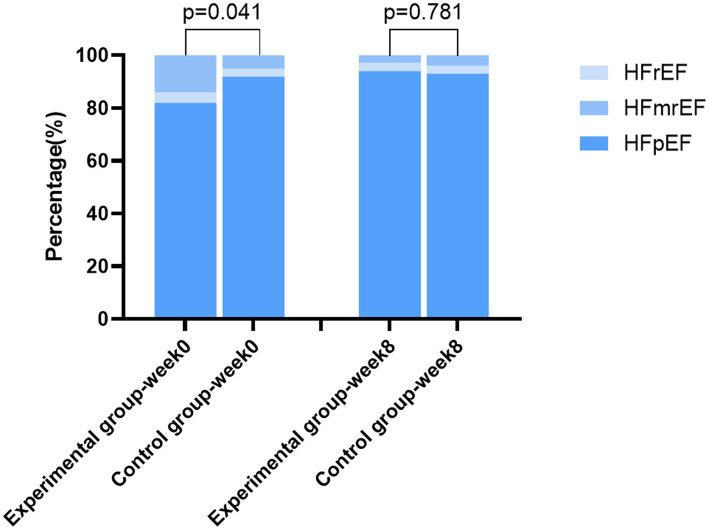
Stacked bar chart of LVEF distribution in the two groups. Each bar represents the percentage of patients in different LVEF categories. Differences between groups were analyzed using the Wilcoxon rank-sum test.

We further compared the distribution of HFrEF, HFmrEF, and HFpEF subtypes between the two groups at baseline and post-treatment. At baseline, the distribution of HF subtypes differed significantly between groups (*z* = −0.153, *p* = 0.041). Specifically, the experimental group had a higher proportion of patients with HFrEF and HFmrEF, suggesting a more severe baseline HF phenotype compared with the control group. Following treatment, no significant between-group difference was observed in the subtype distribution (*z* = −7.235, *p* = 0.781). Details are presented as a stacked bar chart in Figure 4.

These results indicate that both treatments improve cardiac systolic function, and the experimental treatment offers a greater benefit in improving LVEF.

### 3.5 Effects on MLHFQ

At baseline, the MLHFQ scores were 64.55 ± 14.45 points in the experimental group and 60.86 ± 17.97 points in the control group, with no significant difference between groups (*p* = 0.111). After treatment, MLHFQ scores were significantly reduced relative to baseline in both groups (*p* < 0.05). The experimental group had a final score of 49.72 ± 13.44 points, whereas the control group scored 58.24 ± 19.26 points, showing a statistically significant between-group difference (*p* < 0.001; Table 5). After adjusting for age, gender, NYHA functional class and LVEF subgroup distribution, the intervention remained independently associated with a significant improvement in the total TCM syndrome score (*B* = 7.812, 95%CI: 3.581 to 12.042, *p* < 0.001). These findings demonstrate that both interventions alleviate HF-related symptoms and improve quality of life, with a more pronounced effect in the experimental group.

### 3.6 Effects on 6MWD

Baseline 6MWD was comparable between the experimental group 277.19 ± 74.29 meters and the control group 265.73 ± 58.92 meters (*p* = 0.228). After treatment, 6MWD increased significantly in both groups (*p* < 0.05), reaching 457.38 ± 99.66 meters in the experimental group and 383.66 ± 120.91 meters in the control group. Notably, the between-group difference in post-treatment 6MWD was statistically significant (*p* < 0.001; Table 5). After adjusting for age, gender, NYHA functional class and LVEF subgroup distribution, the intervention remained independently associated with a significant improvement in the total TCM syndrome score (*B* = −73.899, 95%CI: −105.177 to −42.621, *p* < 0.001). This suggests that both treatments enhance exercise tolerance in patients with CHF, and the experimental treatment leads to a greater improvement in exercise capacity.

### 3.7 Effects on RAAS

RAAS data were available for 56 patients, with 28 patients in each group. Owing to its non-normal distribution, PRA was logarithmically transformed (ln-PRA) before statistical analysis; after log-transformation, the data conformed to a normal distribution. Ang II and ALD were normally distributed and thus analyzed directly.

At baseline, there were no significant between-group differences in plasma PRA, Ang II, or ALD levels (*p* > 0.05). Compared with baseline, plasma levels of PRA, Ang II, and ALD were all significantly reduced in the experimental group after treatment (*p* < 0.05). In the control group, only PRA was significantly decreased (*p* < 0.05), whereas Ang II and ALD levels showed no significant changes (*p* > 0.05). After treatment, plasma levels of PRA, Ang II, and ALD in the experimental group were all significantly lower than those in the control group (*p* < 0.05; Table 7). These results indicate that the experimental treatment can comprehensively suppress the over activation of the RAAS in patients with CHF, whereas the control treatment only reduces PRA without affecting Ang II and ALD levels.

**Table 7:** Outcomes of RAAS before and after treatment in two groups.

Subject	Time point	Experimental group	Control group	*t*	*p*
ln-PRA (ng/mL)	Week 0	0.38 ± 0.96	0.59 ± 1.13	0.759	0.451
	Week 8	0.91 ± 1.17^*^	0.11 ± 0.84^*^	−2.939	0.005^#^
Ang II (pg/mL)	Week 0	65.67 ± 17.63	67.48 ± 12.56	−0.444	0.659
	Week 8	51.80 ± 14.52^*^	67.00 ± 12.85^**^	−4.149	<0.001^#^
ALD (pg/mL)	Week 0	83.48 ± 61.35	73.21 ± 43.99	0.719	0.475
	Week 8	51.44 ± 34.40^*^	75.72 ± 52.71^**^	−2.042	0.046^#^

*Comparison between Week 8 and Week 0 within the same group, *p* < 0.05; **Comparison between Week 8 and Week 0 within the same group, *p* > 0.05; #*p* < 0.05.

### 3.8 Effects on NYHA functional class

**Figure 5:**
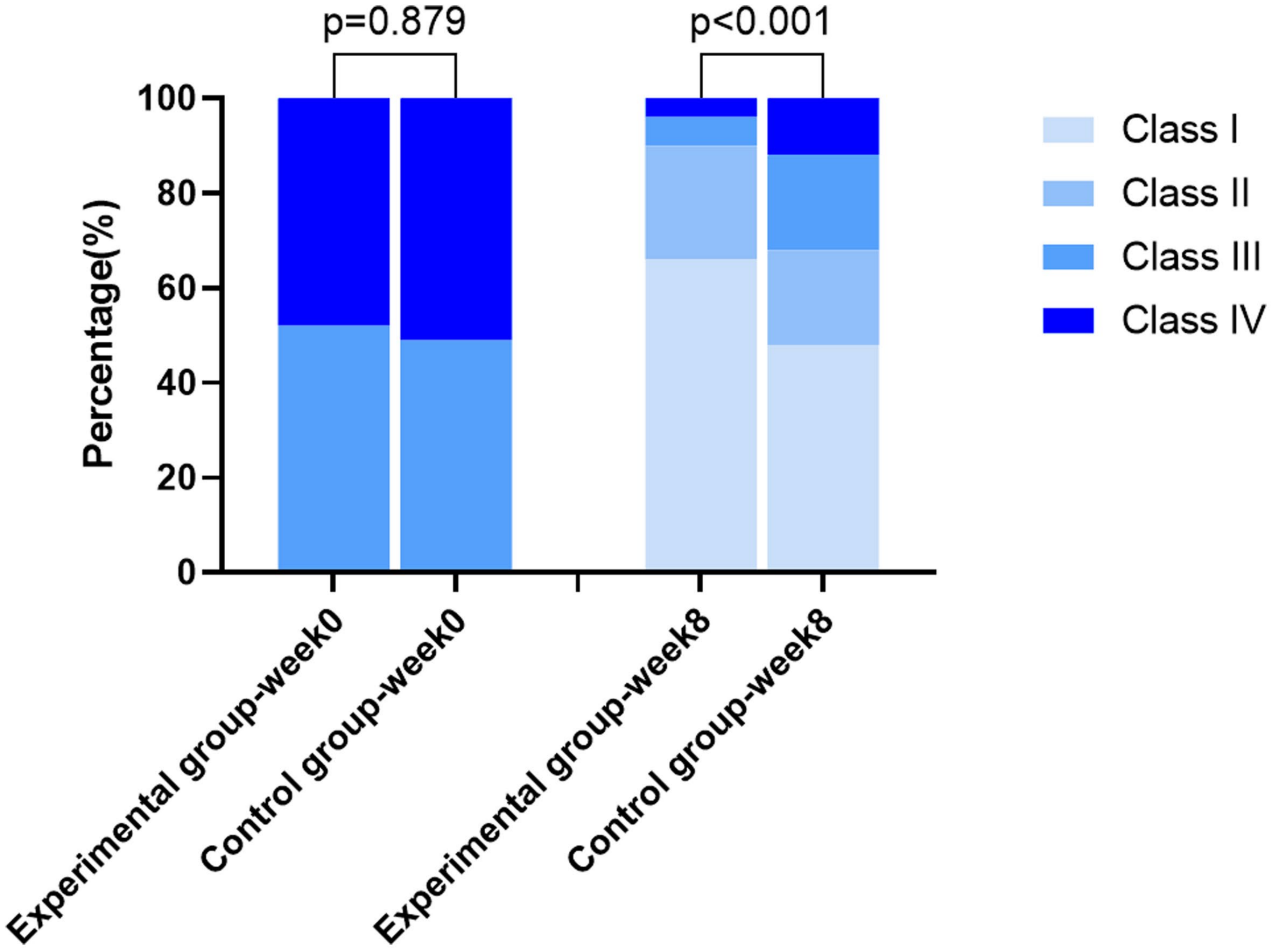
Percentage distribution of patients according to NYHA functional class. Each bar represents the percentage of patients in different NYHA functional classes. Differences between groups were analyzed using the Wilcoxon rank-sum test.

Before treatment, there was no significant difference in NYHA functional class between the experimental and control groups (*p* = 0.879). After treatment, the experimental group showed significantly greater improvement in NYHA functional class compared with the control group (*p* < 0.001). Details are presented as a stacked bar chart in Figure 5. These findings indicate that the experimental treatment provides a significantly better improvement in cardiac functional class in patients with CHF.

### 3.9 MACE

No deaths occurred during the treatment or the subsequent 3-month follow-up period.

**Figure 6:**
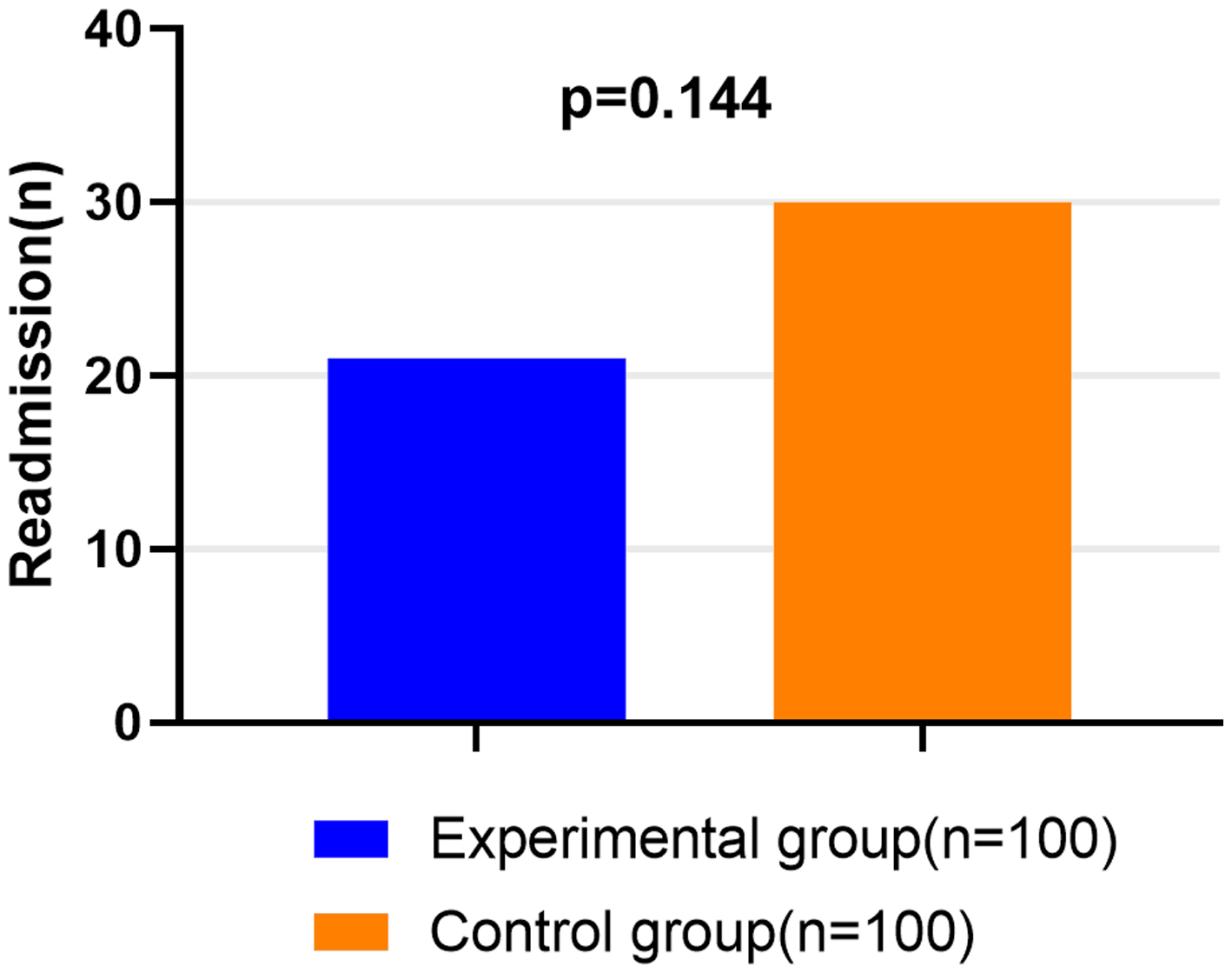
Comparison of the number of 3-month readmissions between groups. Each bar represents the number of readmission cases within 3 months. Differences between groups were analyzed using the Wilcoxon rank-sum test.

Therefore, the MACE outcome was equivalent to HF-related readmission. During 3-month follow-up, 21 patients in the experimental group and 30 patients in the control group were readmitted for HF. The between-group difference was not statistically significant (21% vs. 30%, *χ*^2^ = 2.132, *p* = 0.144). These results confirm a zero 3-month mortality in both groups, and show that the two treatments have similar short-term effects on HF-related readmission. The results are presented as a bar chart in Figure 6.

### 3.10 Safety

No adverse events were observed in either group. Throughout the study, no participants discontinued the trial due to local injection-site reactions, procedural pain, or any systemic adverse events. All previously reported dropouts were unrelated to safety concerns or intervention-related intolerance. No significant changes in AST levels were observed in either group after treatment compared with baseline (*p* > 0.05). In the experimental group, CRE levels decreased significantly from 89.26 ± 39.60 μmol/L to 82.05 ± 33.39 μmol/L (*p* < 0.05), whereas no significant change in CRE was found in the control group (*p* > 0.05). After treatment, there were no significant between-group differences in AST or CRE levels (*p* > 0.05; Table 8). These results suggest that both treatments are safe and do not cause obvious impairment of hepatic or renal function.

**Table 8:** Safety indicators before and after treatment between the two groups.

Subject	Time point	Experimental group	Control group	*t*	*p*
AST (U/L)	Week 0	28.71 ± 14.94	30.70 ± 28.21	−0.624	0.533
	Week 8	27.25 ± 12.72^**^	29.24 ± 15.87^**^	−0.978	0.329
CRE (μmol/L)	Week 0	89.26 ± 39.60	88.24 ± 32.12	0.201	0.841
	Week 8	82.05 ± 33.39^*^	85.45 ± 36.65^**^	−0.687	0.493

*Comparison between Week 8 and Week 0 within the same group, *p* < 0.05; **Comparison between Week 8 and Week 0 within the same group, *p* > 0.05.

## 4 Discussion

In the present study, we investigated the clinical efficacy and safety of acupoint injection of astragalus injection at PC6 combined with conventional treatment in patients with CHF by assessing cardiac function, quality of life, exercise tolerance, TCM syndromes, neurohumoral activation, and short-term clinical events. The results demonstrated that compared with the control treatment, the experimental treatment further reduced TCM syndrome scores, BNP, MLHFQ scores, plasma PRA, Ang II and ALD levels, increase LVEF and 6MWD, and improve NYHA functional class. No deaths occurred in either group during the 3-month follow-up, and no significant between-group difference was observed in HF-related readmission rate. Collectively, these findings indicate that acupoint injection of astragalus injection at PC6 is safe and effective for the treatment of CHF, providing superior benefits in cardiac function, clinical symptoms, and neurohumoral regulation.

Our findings confirm our previous hypothesis that acupoint injection of astragalus injection at PC6 significantly alleviates TCM symptoms in CHF patients with qi-deficiency and blood-stasis syndrome, including wheezing, fatigue, palpitations, lassitude and reluctance to speak, spontaneous sweating, low voice and purplish lips. Subgroup analysis showed that the therapeutic effect was statistically significant in all stratified subgroups. Meanwhile, no significant interaction was observed across subgroups, indicating that the research conclusion was robust and stable. The outcome was not affected by baseline characteristics such as gender, age, BNP, HF type, NYHA functional class, hypertension and diabetes.

Subgroup analyses for BNP, stratified by LVEF revealed distinct statistical outcomes across subgroups, which were highly correlated with the imbalanced sample size distribution. This phenomenon can be primarily attributed to the insufficient statistical power in the two LVEF-reduced subgroups with small sample sizes. Small sample volumes are prone to type II statistical errors, which may mask potential inter-group differences and result in non-significant *p* values despite possible clinical discrepancies. Furthermore, the severely skewed case allocation in this study reflects the inherent epidemiological characteristics of the target clinical population, where patients with preserved cardiac function account for the vast majority, while those with moderate-to-severe LVEF reduction are relatively rare in clinical practice. Given the above limitations, the subgroup findings in the reduced LVEF populations should be interpreted as exploratory rather than definitive conclusions. Future large-scale, multi-center clinical studies are warranted to enroll more patients with mildly and moderately reduced LVEF, balance the sample size across subgroups, improve the statistical test efficiency, and further validate the generalizability of the present results.

Dysregulation of the RAAS is postulated to be significant regulators of cardiovascular function, thereby playing a pivotal role in its pathophysiology. The RAAS is frequently hyperactive in HF, which increases fluid retention and worsens cardiac function (37). Our study also demonstrates that acupoint injection of astragalus injection at PC6 significantly reduces plasma levels of PRA, Ang II and ALD in CHF patients. The efficacy of this treatment may be related to its suppression of excessive RAAS activation.

The occurrence and progression of CHF with qi-deficiency and blood-stasis syndrome may be associated with TGF-*β*1 (38). Astragalus has been reported to regulate the TGF-*β*/Smad signaling pathway (39). These observations may provide a theoretical foundation for our research. However, no direct evidence has yet linked the therapeutic effects of PC6 acupoint injection with astragalus in this patient population to the TGF-*β*/Smad pathway.

Research has demonstrated that astragalus can downregulate the expression of inflammatory cytokines such as TNF, IL-1*β*, and IL-6, inhibit the NF-κB and MAPK signaling pathways, thereby alleviating the excessive activation of the RAAS (21). In addition, the PI3K/AKT signaling pathway is also a well-studied therapeutic target. For example, Li et al. (2025) indicated that, astragalus injection can activate mitophagy and protect mitochondrial function in CHF via inhibiting PI3K/AKT/mTOR pathway, and ultimately achieve the therapeutic effect of improving cardiac function (40). A study integrating network pharmacology analysis, molecular docking technology, and *in vitro* experimental validation confirmed that astragalus membranaceus can effectively mitigate ischemic HF through activating PI3K-AKT signaling pathway. This finding aligns with the results of Bai et al. (2025), who proposed that *Astragalus polyphenols* attenuates doxorubicin-induced cardiotoxicity by activating the PI3K/AKT/NRF2 pathway (41).

Meanwhile, a network pharmacological analysis revealed that five active ingredients of *Astragalus membranaceus* including isorhamnetin, Quercetin, Calycosin, Formononetin, and Kaempferol might be the primary active ingredients of astragalus, dominating its cardioprotective effects against HF through regulating ESR1 expression (14). These five active ingredients can partially mitigate the adverse effects of Ang II on the heart, such as slowing myocardial cell hypertrophy and downregulating ANP, BNP, *β*-MHC, and CTGF expressions. Simultaneously, they can also attenuate inflammation in Ang II-induced cardiomyocytes by inhibiting the levels of TNF-*α*, IL-1*β*, IL-18, and IL-6. Similarly, Dai et al. (2023) proposed that astragalus improves ventricular remodeling via ESR1 downregulation RhoA/ROCK pathway (11). Another study also analyzed the mechanism of quercetin in CHF, with results showing that quercetin mitigates ventricular remodeling and myocardial fibrosis through Akt/Bcl-2 pathway activation (42). Clearly, these two studies reached differing conclusions regarding the mechanism of action of the same active component in astragalus in treating CHF.

Some studies have also proposed other theories. For example, astragalus injection can inhibit the apoptosis of H9c2 cardiomyocytes induced by Ang II, and its mechanism may be related to the regulation of the expression of MAM structural proteins and the influence of intracellular calcium balance. Astragalus may treat HF through the above mechanisms (43, 44).

In summary, the mechanism by which astragalus injection administered via acupoint injection at PC6 treats CHF may be related to the suppression of excessive RAAS activation, potentially achieved through the following pathways: (1) Blocking the binding of angiotensin II (Ang II) to the type 1 receptor (AT1R). (2) Inhibits the activation of the MAPK signaling pathway by Ang II-AT1R, which promotes cardiac hypertrophy. (3) Inhibits the activation of the TGF-*β*/Smad signaling pathway by Ang II-AT1R, which promotes myocardial fibrosis. Simultaneously, it suppresses the expression of CTGF, a key downstream effector of TGF-*β*. (4) Inhibits the activation of the NF-κB signaling pathway by Ang II-AT1R, thereby downregulating the expression of certain inflammatory cytokines, such as TNF, IL-1*β*, IL-6, IL-18. (5) Inhibits cardiomyocyte apoptosis by modulating the PI3K/AKT signaling pathways (AKT/mTOR, AKT/NRF2, Akt/Bcl-2).

Our findings are in agreement with the work of Wang et al. (2024), who indicates that intravenous administration of astragalus injection can improve quality of life, enhance exercise tolerance, and increase LVEF in patients with CHF presenting with qi-deficiency and blood-stasis syndrome (45). However, the treatment in this study involved intravenous administration of astragalus injection rather than acupoint injection at specific points, which differs from our research. Another study with findings similar to ours is that of Wu et al. (2020), who also observed that acupuncture at PC6 improves quality of life and cardiac function in patients with CHF, alleviating symptoms such as palpitations, chest tightness, and dyspnea (31). The mechanism may involve downregulating TGF-*β*1, reducing expression of the fibrotic growth factor, and thereby repairing cardiac function. However, this study only employed acupuncture at PC6 without adjunctive acupoint injection of astragalus injection. Although some studies have combined acupoint injection with astragalus injection, the acupoint selected in these studies was Zusanli (ST36), not PC6. Moreover, previous relevant studies were limited by outdated design and small sample sizes, while the present study provides updated evidence with more rigorous methodology.

In contrast to previous reports, we found that acupoint injection of astragalus injection at PC6 did not significantly reduce the 3-month hospital readmission rate in patients with CHF compared with conventional standard therapy alone. Several potential explanations may account for the divergent findings. First, the 3-month follow-up period in this study was relatively short to capture the potential delayed protective effects of astragalus, which is characterized by gradual Qi-tonifying and heart-nourishing actions in TCM theory. Second, hospital readmission represents a comprehensive endpoint influenced by multiple factors, including comorbidities, medication adherence, sodium and volume management, socioeconomic status, and access to healthcare, which may dilute the measurable effect of a single adjuvant acupoint therapy. Furthermore, differences in study design, sample size, baseline disease severity, and frequency of acupoint administration may also contribute to the inconsistent results. Collectively, our findings indicate that acupoint injection of astragalus at PC6 is not superior to standard therapy in reducing short-term hospital readmission in CHF patients, but does not negate its potential value in improving clinical symptoms and functional capacity. Future studies with longer follow-up, optimized intervention protocols, larger sample sizes, and composite cardiovascular endpoints are warranted to further clarify the role of this integrative therapy in CHF management.

It should be noted that our study has several limitations. First, the sample size for RAAS analysis was relatively small, which may reduce statistical power. Nevertheless, calculation based on our preliminary data confirmed that 56 subjects (28 per group) satisfied the minimal statistical requirement (22). Second, qi-deficiency and blood-stasis syndrome mainly occurs in mild CHF, consistent with previous observations (46). However, from the perspective of syndrome research, this distribution pattern also lends support to the views of other researchers (46). Third, 87% of patients had HFpEF, 3.5% had HFrEF, and 9.5% had HFmrEF. The limited number of HFrEF and HFmrEF patients precluded reliable subgroup analyses to assess the influence of LVEF on treatment response.

Thus, our findings are primarily applicable to CHF patients with qi-deficiency and blood-stasis syndrome; generalization to other TCM syndromes requires further validation. Future studies should perform prospective, multi-syndrome, stratified randomized trials to compare efficacy across TCM patterns. More research focusing on severe TCM syndromes and dynamic changes in syndrome patterns during disease progression is also needed. This will help establish a more comprehensive evaluation system for PC6 acupoint injection with astragalus injection.

Furthermore, while we demonstrated that this treatment inhibits excessive RAAS activation in CHF, its precise downstream signaling pathways remain unclear. Future mechanistic studies are warranted to clarify the molecular mechanisms underlying RAAS regulation, especially in the disease-syndrome model of qi-deficiency and blood-stasis CHF. Longer follow-up durations are also needed to confirm the long-term efficacy and safety of this approach.

This study demonstrates that acupoint injection of astragalus injection at PC6 combined with conventional treatment can significantly relieve HF-related symptoms, and enhance quality of life and exercise capacity in CHF patients with qi-deficiency and blood-stasis syndrome. The underlying mechanism may be related to the inhibition of excessive RAAS activation, thereby suppressing the Ang II-AT1R-mediated activation of downstream signaling pathways. In addition, the combined treatment exhibited favorable hepatic and renal safety, suggesting that it is a safe and effective adjuvant treatment for CHF.

## Data Availability

The raw data supporting the conclusions of this article will be made available by the authors, without undue reservation.
