# Correction: Effect and safety of Neiguan acupoint injection with astragalus injection for chronic heart failure with qi-deficiency and blood-stasis syndrome: a randomized controlled trial

**DOI:** 10.3389/fmed.2026.1902914

**Published:** 2026-07-15

**Authors:** Dongmei Gou, Maomei He, Jiaquan Zhuo

**Affiliations:** Department of Cardiology, Traditional Chinese Medicine Hospital of Renshou County, Renshou, China

**Keywords:** astragalus injection, chronic heart failure, Neiguan acupoint, qi-deficiency and blood-stasis syndrome, renin-angiotensin-aldosterone system

The funder Traditional Chinese Medicine Special Project of the Third Batch of 2023 Municipal Guiding Science and Technology Programs, Meishan Municipal Bureau of Science and Technology (No. ZYY202309) to Dongmei Gou, Maomei He and Jiaquan Zhuo was erroneously omitted. The correct **Funding** statement reads:

The author(s) declared that financial support was received for this work and/or its publication. This research was funded by the 2024 Special Research Project of Traditional Chinese Medicine of Sichuan Provincial Administration of Traditional Chinese Medicine (No. 2024MS430) and Special Scientific Research Project of Sichuan Medical Science and Technology Innovation Research Association (No. YCH-KY-YCZD2024-220). Traditional Chinese Medicine Special Project of the Third Batch of 2023 Municipal Guiding Science and Technology Programs, Meishan Municipal Bureau of Science and Technology (No. ZYY202309) to Dongmei Gou, Maomei He, and Jiaquan Zhuo.

The original version of this article has been updated.

